# Bariatric surgery in adolescents: a prospective randomized controlled trial comparing laparoscopic gastric banding to combined lifestyle interventions in adolescents with severe obesity (BASIC trial)

**DOI:** 10.1186/s12887-019-1395-9

**Published:** 2019-01-28

**Authors:** Y. G. M. Roebroek, G. F. Paulus, E. G. A. H. van Mil, A. C. E. Vreugdenhil, B. Winkens, C. Nederkoorn, C. D. A. Stehouwer, J. W. M. Greve, N. D. Bouvy, L. W. E. van Heurn

**Affiliations:** 1 Department of General Surgery, Maastricht University Medical Centre, P.O. Box 5800, NL-6202 AZ Maastricht, The Netherlands; 2 NUTRIM School of Nutrition and Translational Research in Metabolism, Maastricht University, Maastricht, The Netherlands; 3 Department of General Surgery, Spaarne Gasthuis, Haarlem, The Netherlands; 4 Department of Paediatrics, Jeroen Bosch Medical Centre, ‘s Hertogenbosch, The Netherlands; 5 Department of Paediatrics, Maastricht University Medical Centre, Maastricht, The Netherlands; 6 Department of Methodolgy and Statistics and CAPHRI Care and Public Health Research Institute, Maastricht University, Maastricht, The Netherlands; 7 Department of Clinical Psychological Science, Maastricht University, Maastricht, The Netherlands; 8 Department of Internal Medicine and CARIM School for Cardiovascular Diseases, Maastricht University, Maastricht, The Netherlands; 9 Department of General Surgery, Zuyderland Medical Centre, Heerlen, The Netherlands; 10 Department of Pediatric Surgery, Emma Children’s Hospital, Amsterdam Medical Centre/ VU University Medical Centre, Amsterdam, The Netherlands

**Keywords:** Severe obesity, Adolescents, Bariatric surgery, Safety, Weight loss

## Abstract

**Background:**

Obesity in children and adolescents is an increasing problem associated with multiple co-morbidities including metabolic and endocrine changes, cardiovascular abnormalities, and impaired quality of life. Combined lifestyle interventions are the current standard treatment for severe obesity in children. However, the medium- and long-term results of these interventions are relatively poor. Bariatric surgery shows substantial weight loss and health improvement in adults and retrospective studies in adolescents show similar outcomes. However, well-designed prospective studies in this young age group are rare. Our objectives are to determine whether combining surgery with lifestyle interventions in severely obese adolescents leads to a significant additional weight reduction compared to lifestyle interventions solely, and to assess its effect on obesity-associated co-morbidities in a prospective randomized controlled setting.

**Methods:**

Patients aged 14–16 years with sex- and age-adjusted BMI > 40 kg/m^2^ (or > 35 kg/m^2^ with comorbidity) and failure to achieve weight reduction > 5% during at least one year of combined lifestyle interventions are included in this trial. Randomization determines whether laparoscopic adjustable gastric banding will be added to combined lifestyle intervention throughout the trial period. Sixty children will be included in this trial. Follow-up visits are planned at 6 months, 1,2 and 3 years. Primary endpoints are percentage of total weight loss, and change of BMI. Secondary endpoints include body composition, pubertal development, metabolic and endocrine changes, inflammatory status, cardiovascular abnormalities, non-alcoholic steatohepatitis, quality of life and changes in behaviour.

**Discussion:**

This randomized controlled trial is designed to provide important information about the safety and efficacy of laparoscopic adjustable gastric banding treatment in severely obese adolescents with unsuccessful combined lifestyle interventions. The reversibility of this surgical procedure forms a strong argument to decide for gastric banding over other surgical procedures, since bariatric surgery in adolescents is still in its infancy.

**Trial registration:**

The BASIC trial is registered in the register of ClinicalTrials.gov since July 2010, Identifier: NCT01172899

**Electronic supplementary material:**

The online version of this article (10.1186/s12887-019-1395-9) contains supplementary material, which is available to authorized users.

## Background

Overweight and obesity in children has become a global health problem during the past decades. It has already been designated as ‘one of the most serious public health challenges of the 21^st^ century’ by the World Health Organization [1]. Obesity during childhood can have serious consequences for several organ systems including the cardiovascular and the endocrine, and can result in psychosocial comorbidity [2, 3]. Furthermore, children with overweight and obesity are prone to become obese adults, with an on-going risk of comorbidities [4–6]. If obesity is more severe, or shows a greater variability or rapid increase, adolescents are less likely to grow out of their obesity [4, 7].

In accordance with treatment in adults, there are various treatment methods for overweight and obesity in children. Several interventions have been studied in childhood populations [8–11]. Lifestyle interventions are often based on physical activity, dietary and/or behavioural interventions. Combination of these non-pharmacological lifestyle components can result in a decrease in body mass index (BMI) up to twelve months after treatment, but effect sizes remain small [8]. Evidence for long-term weight control following these lifestyle interventions in children is scarce, but it should be noted that in adults the majority of these interventions have been proven to be ineffective in long-term weight control [12, 13]. Pharmacological treatment can enhance weight loss when prescribed as additional therapy next to lifestyle interventions [8]. Both orlistat and sibutramine seem to be effective in adolescents, but again the effects remain small and long-term data is limited [14–18].

Since the risk of associated comorbidity increases with an increasing BMI, the need for an effective and long-term solution to lose weight is even more urgent for children and adolescents with severe obesity [19, 20]. In the adult population, bariatric surgery is widely accepted as treatment for severe obesity. Bariatric surgery enables patients to lose more weight compared to non-surgical interventions, regardless of the type of surgery [21]. In addition, the effect of bariatric surgery has been proven to persist over many years, leading to reduction in mortality and obesity-associated comorbidity in the long-term [21–23]. Because of ethical considerations and the potential for serious complications, bariatric surgery is not generally accepted (yet) as a last-resort treatment option in therapy-resistant severely obese children. In order to substantiate the additional value of bariatric surgery compared to treatment with lifestyle interventions alone, more evidence on safety and effectiveness of childhood bariatric surgery is required. Few prospective cohort studies on childhood bariatric surgery have been published. The majority of these studies address one of the three common bariatric procedures: laparoscopic adjustable banding (LAGB), laparoscopic sleeve gastrectomy (LSG), and laparoscopic roux-and-y gastric bypass (LRYGB) [24, 25]. In general, these cohort studies suggest effective weight loss up to 36 months (− 10 to − 15 BMI points depending on the procedure) [26]. However, only one randomized controlled trial has been carried out to compare bariatric surgery with conventional treatment in adolescents, resulting in − 12.7 versus − 1.3 BMI points after 2 years for intervention and control group respectively [27]. The present study differs from this previous study, as we include only patients who underwent lifestyle interventions in a multidisciplinary setting before for more than 1 year and nevertheless failed to lose weight [10, 11].

Despite the lack of the highest quality evidence that bariatric surgery in adolescents is safe and can be successfully done, this treatment has been popularised in certain countries to treat adolescents with severe obesity. In most countries is in Europe, they are more reluctant to do an operation for severe obesity, or bariatric surgery in youngsters is not performed at all.

### Study objectives

Combined Lifestyle Interventions (CLI) are the current standard to obtain weight reduction in severely obese children and adolescents [10, 28, 29]. The main aim of the ‘Bariatric Surgery in Children’ (BASIC) randomized controlled trial is to investigate whether last-resort LAGB treatment in children and adolescents is safe and effective in terms of (excess) weight loss and loss of excess BMI. Secondary objectives in this trial focus on obesity associated comorbidity and include assessment of body composition, pubertal development, metabolic and endocrine changes, inflammatory status, cardiovascular abnormalities, non-alcoholic fatty liver disease, quality of life, general and food-specific cognitive processes, changes in behaviour and effects on sleep architecture.

## Methods

### Study design

We will perform a prospective superiority trial by randomizing severely obese children and adolescents in a 1:1 ratio to receive LAGB on top of the CLI versus CLI treatment alone. The study protocol meets the criteria of the ‘Standard Protocol Items: Recommendations for Interventional Trials’ (SPIRIT) Guidelines (Additional file 1: Appendix 1) [30]. The trial will be carried out at the Maastricht University Medical Centre in the Netherlands. Outcomes will be measured at six, 12, 24 and 36 months from randomisation and will be evaluated after 12 and 36 months.

### Study population

#### Inclusion criteria

The study population includes severely obese children who underwent long-term multidisciplinary lifestyle interventions without the intended effect. All children referred to our clinic for enrolment in the trial are screened for the following inclusion criteria: age 14, 15 or 16 years old; age- and sex-adjusted BMI > 40 kg/m^2^ or > 35 kg/m^2^ with obesity associated co-morbidity (Table 1) [29, 31]; and participation in at least one multidisciplinary organized paediatric weight reduction program (as combined lifestyle interventions, CLI) for a minimum period of 12 months, without accomplishing the intended effect of 5% weight loss or more. The CLI should be coordinated and monitored by a paediatrician and include at least regular dietary advice and monitoring from a certified dietician, regular exercise training and behavioural therapy from a trained psychosocial worker.

**Table 1:** Age- and sex-adjusted BMI cut-off points for severely obesity in the Dutch population [29]

	Boys		Girls	
Age [years]	BMI 35 [kg/m^2^]	BMI 40 [kg/m^2^]	BMI 35 [kg/m^2^]	BMI 40 [kg/m^2^]
14	32.9	38.4	33.3	39.4
15	33.7	39.1	33.9	39.7
16	34.2	39.5	34.3	39.9

Obesity-associated co-morbidity in this young population has been defined as one or more of the following: a) glucose intolerance (fasting glucose > 6.1 mmol/L); type 2 diabetes mellitus (T2DM; fasting glucose > 7.0 mmol/L and/or oral medication on prescription for known T2DM); b) hypertension (blood pressure > 140 mmHg systolic and/or > 90 mmHg diastolic, and/or oral medication on prescription for known hypertension); c) dyslipidaemia (total cholesterol > 6.5 mmol/l, low-density lipoprotein cholesterol > 4.4 mmol/l, high-density lipoprotein cholesterol < 0.9 mmol/l, triglycerides > 1.94 mmol/l and/or oral medication on prescription for known dyslipidaemia); d) obstructive sleep apnoea (OSA; apnoea-hypopnoea index > 5/h, or continuous positive airway pressure treatment for known OSA); e) non-alcoholic steatohepatitis (serum alanine aminotransferase > 45 U/L for males and > 35 U/L for females, or steatohepatitis proven by biopsy); f) depressive disorder (as diagnosed according to the DSM-IV or DSM-V) [32]; g) arthropathy; acanthosis nigricans; and/or idiopathic intracranial hypertension.

Only the comorbidities diagnosed and reported by the treating paediatrician and/or general practitioner of the patient are noted during assessment of inclusion criteria. The participation in one or more multidisciplinary weight reduction programs for a minimum period of 12 months needs to be confirmed by the referring physician.

In addition to the obesity-related inclusion criteria, patients will need to demonstrate an adequate level of decisional capacity, a sufficient level of Dutch language comprehension and awareness of the randomization process with all its consequences; this will be assessed by an independent specialised youth psychologist during an intake session.

#### Exclusion criteria

The exclusion criteria for this trial are a) eating disorders (‘binge eating’, defined as compulsive overeating or consuming abnormal amounts of food while feeling unable to stop and at loss of control’ and ‘grazing’, defined as repetitive periods of eating small amounts of food in an unplanned manner, at least twice daily); b) unrealistic expectations of weight loss; and inadequate social support of the family or caregivers, all to be assessed by the independent specialised youth psychologist and dietician. Dutch translations of validated questionnaires will be used for the assessment of the specific eating disorder exclusion criteria (i.e. the Eating Disorders Examination Questionnaire for Children, chEDE-Q); c) skeletal or developmental immaturity (premenarchal girls, boys with bone age younger than 15 years on X-ray of the hand); d) severe cardiorespiratory impairment (ASA class 3 or more); and e) syndromic disorders causing obesity (e.g. Prader-Willi syndrome). Other exclusion criteria are physical disorders with highly possible influence on weight, such as untreated hypothyroidism or prolactinoma; and unwillingness to adhere to the follow-up program.

### Study outline

**Fig. 1:**
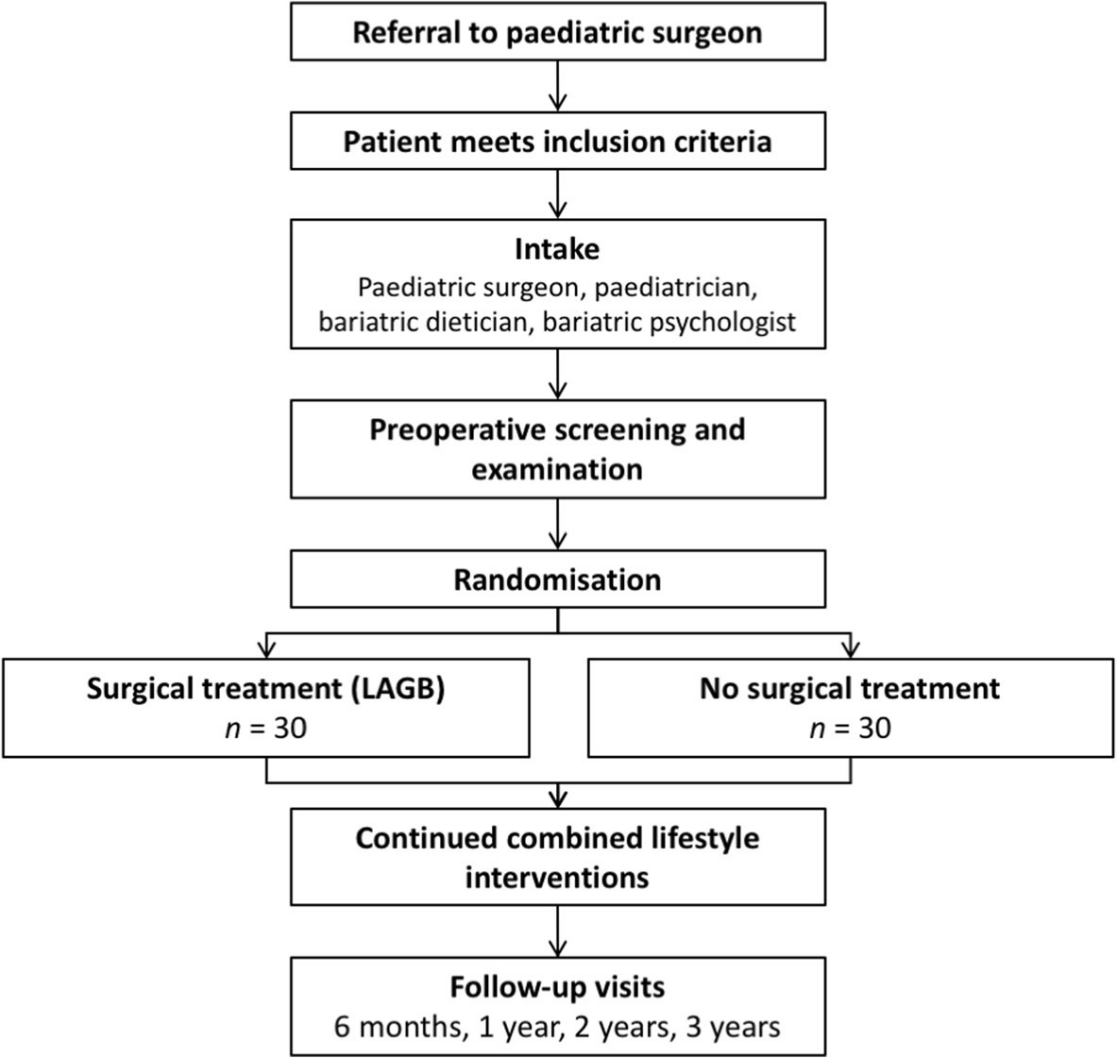
Flowchart of the BASIC trial study design

A paediatrician or general practitioner can refer adolescents to the BASIC trial research team at Maastricht University Medical Centre. The referring physician will report general background of the patient, physical status and previous attempts to reduce weight including lifestyle interventions (among others). If the inclusion criteria on age, BMI and lifestyle interventions are met, the patient and his/her parents will be contacted and invited for an extensive intake at our medical centre. The surgeon, paediatrician, bariatric dietician, paediatric psychologist and clinical investigator (MD) from the BASIC trial research group will carry out the intake session subsequently (Fig. 1).

During the intake session, the patient and his/her parents or legal guardians will be extensively informed about the various aspects of the trial. A routine physical examination will be carried out to make sure all the inclusion criteria are met. Boys will undergo assessment of their bone age with an X-ray of their left hand; an experienced paediatric radiologist will assess the exact bone age. The bariatric dietician and the paediatrician will assess the quality of the attempts to lose weight and acquire possible contra-indications for enrolment in the trial. The patient will be asked about his or her motivation to enter the trial and the expectations of the surgical procedure and its consequences. The motivation, basic understanding of the possible operation and the expectations will be written down by the patient to verify if he or she has understood which decision is to be made. If patients continue to have too optimistic expectations about surgery, or do not understand the implications and consequences of the operation, they will not be considered as suitable candidates to participate in the trial. The expert bariatric psychologist will advise both the patient and the parents.

Afterwards, the patient will be discussed in a team meeting of paediatric and bariatric surgeons, bariatric dietician, paediatrician and paediatric psychologist. If one or more team members object to inclusion of patient, the patient will not be included in the trial and will be referred back to the referring paediatrician or general practitioner.

If the patient is considered suitable to participate in the trial, the investigator will obtain written consent from the patient and both parents / legal guardians for trial participation. The patient will then be admitted to the Maastricht University Medical Centre for extensive physical screening. When a patient shows signs or symptoms of syndromic abnormalities or other disorders being a possible cause of obesity (e.g. hypothyroidism), further investigations will be carried out and treatment (other than being bariatric surgery) will be provided by the referring physician. If the physical screening shows no significant abnormalities possibly causing obesity, the patient can proceed to randomization (Table 2).

**Table 2:**
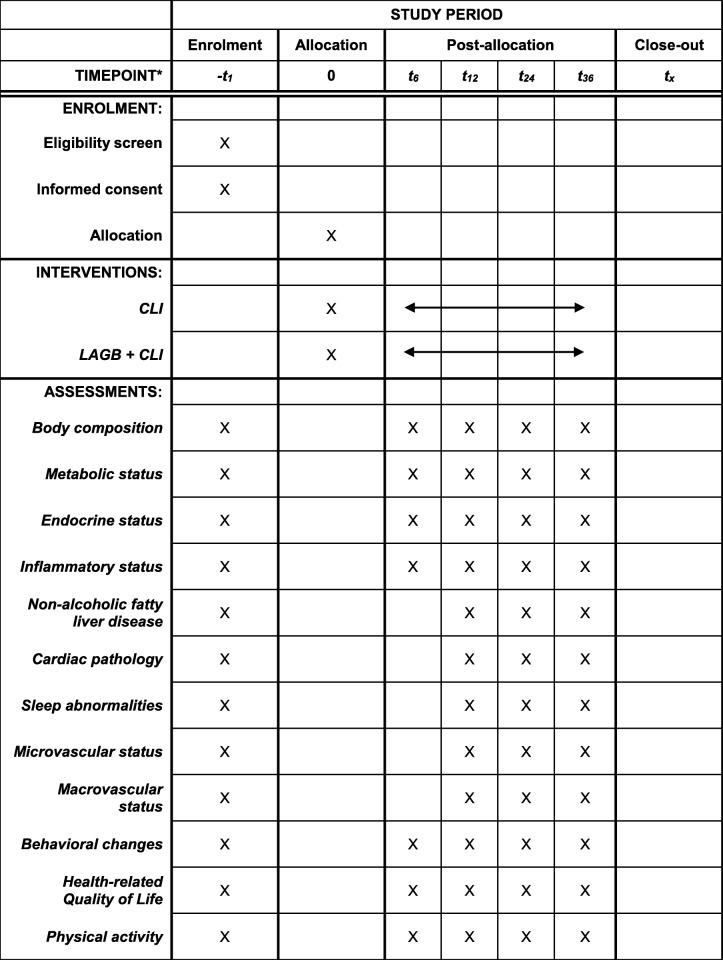
Schedule of enrolment, interventions and assessments of the BASIC trial

*Time points noted in months

*CLI* combined lifestyle interventions, *LAGB* Laparoscopic adjustable gastric banding

The investigator will randomise all patients approved for inclusion in either the ‘surgery and lifestyle group’ (intervention group) or the ‘lifestyle control group’ (control group) at a 1:1 ratio by block randomization. A statistician affiliated with our medical centre will create random blocks using a digital randomization program; the blocks can consist of two, four or six patients. This resulted in 60 sequentially numbered, opaque and sealed envelopes only accessible for principal investigator and paediatric surgeon LvH and for investigator YR. The investigator, bariatric surgeon, paediatric surgeon, patient and his/her parents will not be blinded for randomisation outcome, but will be blinded for the randomisation list. Other care providers from the Maastricht University Medical Centre will be blinded, as will be the data analysts and outcome assessors other than the investigators. Referring paediatricians and general practitioners will not be blinded, to assure adequate care and awareness for potential complications throughout follow-up.

The patients in the intervention group will be readmitted to the surgical centre within 6 weeks after randomisation for routine preoperative evaluation. The dedicated bariatric dietician from the research team will visit the patient and accompanying parents on the ward and provides them with extensive dietary advice and guidelines for the home-based setting. A booklet with summary of the advice will be handed over in duplicate, to make sure the dietician in the CLI treatment team at centre of referral will be provided with the same information. The bariatric surgeon and paediatric surgeon will visit the patient and parents that same day, to reconfirm informed consent for surgery and provide all the information about the surgical procedure, postoperative phase and follow-up. The next day the patient will be operated on under general anaesthesia and receives a LAGB (LAP-BAND AP® Adjustable Gastric Banding System, Allergan Inc., Dublin, Republic of Ireland) using the pars flaccida technique [33]. The expected postoperative hospital stay is one or two days. After 6 weeks, the gastric band can be insufflated gradually if necessary. Adjustments to the volume of the gastric band will be performed at our medical centre and include X-ray control to evaluate the effect of any adjustment, all adjustments will be registered in a standardized file. Afterwards the patient receives yearly routine surgical follow-up, or more often if medically necessary.

All patients participating in the trial (both intervention group and control group) will receive lifestyle interventions and follow-up throughout the entire trial period. All patients will return to the paediatrician, dietician and psychologist of the referring obesity clinic for continuation of the lifestyle program. The multidisciplinary follow-up of the previous life style interventions will be continued in both groups after randomisation to guarantee the best achievable weight loss in both study groups.

The investigator will monitor the post-inclusion care by a telephone follow-up call once every 3 months. All patients will be subjected to an extensive clinical review at the surgical centre at 6 months, 12 months, 2 years and 3 years after inclusion. Adherence to the routine follow-up visits will be registered.

To date, bariatric surgery in children and adolescents is prohibited in The Netherlands for all clinical settings except for this trial. Patients from the control group who turn 18 years during the trial period are offered to crossover to the intervention group and receive surgical treatment. If the patient decides for any other type of weight loss surgery after turning 18, this event will be registered yet the patient will still be invited for follow-up visits and be part in the 3-year follow-up analyses. If this trial will provide sufficient data proving that surgical treatment in adolescents is safe and effective, the Dutch Health Care Inspectorate will evaluate and decide whether the remaining patients from the control group can be offered to crossover to the intervention group.

Patients who withdraw from the trial prematurely will receive their regular follow-up from the referring paediatrician. If patients have been operated upon, they will receive their regular surgical follow-up and treatment, but additional trial related investigations will be discontinued.

### Outcomes

#### Primary outcome measures

These are defined as percentage total weight loss (%TWL) and change in BMI. Height will be noted to the nearest 0.1 cm and body weight to the nearest 0.1 kg, measured with a stadiometer and digital scale respectively with children dressed in underwear. Self-reported weight data will never be used. BMI will be calculated by dividing weight in kilograms by height in meters squared; excess BMI and excess weight will be calculated according to the cut-off points as shown in Table 1.

#### Secondary outcome measures

In addition to height and weight, other anthropometric measurements will be used to assess body composition, including neck, waist and hip circumferences, and skinfold thickness at standardised anatomical points. Dual energy X-ray absorptiometry (DXA) will be used to assess bone density and calculate fat and lean tissue mass. More specific measurement of weight distribution over the different body compartments will be carried out by total body water (TBW) measurement using deuterium dilution [34].

Metabolic and endocrine changes in body homeostasis will be assessed by biochemical analysis of glucose metabolism parameters (both fasting and during oral glucose tolerance test), lipid metabolism parameters (cholesterol, free fatty acids, and triglycerides), thyroid function parameters (thyroid stimulating hormone, free T4, parathyroid hormone) and gonadotropins. Decreased insulin sensitivity (insulin resistance) will be measured using the homeostatic model assessment of insulin resistance (HOMA-IR), while glucose intolerance and diabetes will be defined according to the American Diabetes Association [35–37]. Daytime blood pressure will be measured while resting during a period of 60 to 90 min (intervals of 3 min between measurements) using the Mobil-O-Graph ® NG (I.E.M. GmbH, Stolberg, Germany). Hypertension and prehypertension will be defined according to the National High Blood Pressure Education Program update on American guidelines [38]. Inflammatory status will be assessed by biochemical analysis of inflammatory variables.

Liver functioning and potential non-alcoholic hepatitis steatosis will be monitored by testing biochemical liver function variables. Furthermore, a specialised paediatric radiologist will carry out a standardised ultrasound investigation of the liver to screen patients for structural liver abnormalities, monitor liver dimensions and obtain a rough estimate of hepatic steatosis.

Cardiac functioning will be investigated by a specialised paediatric cardiologist, who will perform Doppler echocardiography in each patient yearly, assessing the cardiac dimensions and potential left ventricle hypertrophy. Macrovascular changes will be monitored throughout the trial by measurement of the carotid intima-media thickness, carotid-femoral pulse wave velocity, pulse waveform analysis and flow mediated dilation of the brachial artery [39–41]. Microvascular changes will be measured by testing nailfold capillary density and post occlusive nailfold capillary recruitment [42]. Vasomotion of the microcirculation in the skin will be measured at wrist level during the oral glucose tolerance test using laser Doppler flowmetry [43, 44].

Quality and architecture of sleep and potential obstructive sleep apnoea will be analysed objectively with an annual polysomnography.

Patients will be asked to carry an accelerometer at standardised time points during follow-up. The meter will then be carried for 7 days while performing regular activities. A diary will be kept during these 7 days to gain more insight in the activities. At the same standardised time points patients will be asked to fill out a Baecke questionnaire (adapted for children) evaluating work activity, sports activity and non-sports leisure activity [45].

Eating behaviour and response to food stimuli will be assessed with Dutch translations of validated self-report questionnaires, including the Eating Disorders Examination Questionnaire for Children (chEDE-Q), the Three Factor Eating Questionnaire (TFEQ) and the Power of Food Scale (PFS) [46–48]. A computerized version of the Iowa Gambling Task (IGT) will be used to investigate decision making and reward dominance of the study participants [49, 50]. The Stop Signal Test (SST) will be used to measure response inhibition and monitor potential changes throughout the study [51]. In order to study the rewarding value of food items, the patients will play a Wanting and Liking Test (WLT) before and after a standardised meal [52].

Psychosocial status and functioning will be assessed both at the beginning and throughout the trial. Health-related quality of life (HRQOL) will be monitored with use of the Dutch translation of the Pediatric Quality of Life Inventory (PedsQL) for children aged 13 to 18 years [53]. In addition, the Beck Depression Inventory (BDI)-II will be used to gain more insights in psychosocial functioning [54].

All outcome variables will be measured at baseline, 12 months, 24 months and 36 months. At 6 months, all examinations will be carried out except for DXA, liver and cardiac ultrasound, microvascular and macrovascular changes and the polysomnography.

### Sample size

The total number of needed study participants was calculated for the primary outcome measure weight loss and is based on the following assumptions: (1) the expected weight loss after 1 and 3 years in the surgery group is 25%; (2) the expected weight change in the control group is 0%, as the patients in the control group have previously been unsuccessfully treated with combined lifestyle interventions; (3) the expected standard deviation is 10% for both groups [55]. To assess a difference in weight loss of 10% or more (7% weight loss is considered to be relevant as it results in a significant improvement in risk and co-morbidity in severely obese adolescents), 44 patients have to be included (α = 0.05, β = 0.10) [56]. Based on previous studies in adults at our institution, we assume that approximately 20% of the patients will not finish the one-year follow-up period, 60 patients need to be included into the study to preserve results on 44 patients treated per protocol. The number of patients necessary to show significant relative excess weight loss or loss of relative excess BMI is smaller. The sample size needed to detect significant and relevant weight loss after surgery compared to conservative treatment will also be sufficient to assess significant differences in insulin resistance and hypertension between both groups. The expected resolution of both insulin resistance or diabetes, and hypertension after surgery is 70–90%, while no effect of continuing life style interventions can be expected [55, 57]. For most of the other variables the currently available information about the expected outcome is not sufficient for a reliable power analysis.

### Data collection and management

Standardized case report forms will be used for source data during the trial and stored by the investigator in a locked, fireproof place. To ensure privacy of the participants, identifying data will be stored in coded form secured by a password only known to the investigator and principal investigator. Tissue and plasma samples will be stored in coded form and stored in a locked freezer at the surgical laboratory of the principal investigator. Data and human material will be kept for 15 years. Statistical analyses are done by the professional statistician participating in the research staff of the study. (Additional file 2: Appendix 2).

In order to ensure data quality, both the cardiac and liver ultrasound will be carried out by clinical experts and measurements are repeated at least twice per participant per examination. The DXA outcome variables will be evaluated by two specialised nuclear physicians. Anthropometric measurements and microvascular and macrovascular investigations will be performed by a trained laboratory technician; duplicate measurements will be carried out for all of these specific outcomes.

The results of the study will be presented at scientific meetings and published in peer-reviewed medical journals.

### Safety

Serious adverse events and adverse events will be registered in accordance to the standards of Good Clinical Practice. The Dutch Health Care Inspectorate will supervise this clinical trial independently and performs unannounced audits. All early and late operative complications such as wound site infections, slippage of the gastric band, and pouch dilatation of the created gastric pouch will be monitored throughout the entire duration of the trial according to the Clavien-Dindo scoring system [58]. Puberty development will be monitored with growth (body height) and plasma levels of gonadotropins and sex hormones. Nutritional status will be assessed to prevent malnutrition.

The study will be terminated prematurely in case of serious and unexpected complications of surgery, if the complication rate of surgery exceeds the 95% confidence interval of the 10% complication rate considered as normal and in case of serious adverse events that are directly related to the diagnostic procedures performed in this study.

### Monitoring

To monitor the safety of all patients included in the trial, a Data Safety Monitoring Board (DSMB) will be installed. The board will consist of three independent physicians from the departments of General Surgery and Paediatrics of the Maastricht University Medical Centre. The progress of the trial will be reported to the DSMB after every tenth inclusion in the trial. The report will contain data on all complications of surgery, all adverse events, major protocol deviations and other relevant information per coded participant. The opinion of the DSMB and consequent binding (dis)approval of continuation of the trial will be written down and stored in the trial master file. The medical ethics committee of Maastricht University Medical Centre will be informed about the DSMB opinions.

## Discussion

The BASIC trial is designed to provide high-quality data on the safety and efficacy of LAGB treatment in severely obese children and adolescents. Lack of available long-term data on safety and efficacy of bariatric surgery in youngsters advocates an extremely cautious approach and intensive monitoring of growth and development in these specific patients. Nevertheless, severe obesity has already proven to impair children and adolescents in their pubertal and psychosocial development and significantly increases risks for development of cardiovascular diseases amongst others [2–6]. The BASIC trial will provide tertiary and multidisciplinary care for its participants: comprehensive health examinations will be carried out throughout the trial and results will be discussed in a multidisciplinary setting to guarantee optimal care for this vulnerable population. This is the first randomized controlled trial investigating bariatric surgery as a complementary treatment in adolescents with failed combined lifestyle interventions. Outcome measurement will focus primarily on percentages of total weight loss and excess weight loss, yet a comprehensive set of secondary outcome measures will be provided as well.

A potential limitation of this study includes the choice of LAGB treatment over other bariatric procedures as investigational intervention. Laparoscopic adjustable gastric banding is a restrictive bariatric procedure that can be reversed easily and enables weight loss without altering absorptive processes. The reversibility of the LAGB procedure formed an important argument in this study design of this experimental trial, since the study population is very young and patients might want to make use of possible new and innovative treatment options in the future. Other bariatric procedures have proven to be more effective in terms of excess weight loss and improvement of comorbidity in adults, though these results do not necessarily apply to children and adolescents in the same extent [21, 25].

Secondly, this study may be at risk for relative high loss-to-follow up rates throughout the study period. Because of the comprehensive attempts to lose weight (without intended effect) prior to enrolment in this study, patients may have developed a certain attitude towards healthcare systems and providers negatively affecting their compliance and their stamina. Allocation to the control group might amplify these developments, resulting in high risks for getting loss-to-follow up. The coordinating investigator of the BASIC trial will be aware of these risks and attempt to keep the patients involved with their own health status and potential beneficial treatment methods. Patients and their parents will be informed of the results of their annual comprehensive medical examination and will be involved in the possible adjustments that have to be made to the combined lifestyle interventions and/or gastric band care if applicable.

In summary, this randomized controlled trial will provide important information on the safety and efficacy of LAGB surgery in severely obese adolescents with unsuccessful CLI. The unique control group will provide a necessary framework of data regarding physiological changes throughout adolescence and pathophysiological effects of persistent childhood severe obesity. The results of this study may steer the international opinion regarding treatment options of severely obese adolescents who still have most of their lives ahead of them.

## Additional files

Additional file 1: Appendix I: SPIRIT Checklist. (PDF 144 kb)

Additional file 2: Appendix II: statistical analysis plan for outcome measurements. (DOCX 12 kb)

## Abbreviations

ASA class: American Society of Anesthesiologists physical status classification
BMI: Body mass index
chEDE-Q: Child version of eating disorders examination questionnaire
CLI: Combined lifestyle interventions
DSMB: Data Safety Monitoring Board
DSM-IV: Diagnostic and Statistic Manual of Mental disorders 4th edition
DSM-V: Diagnostic and Statistic Manual of Mental Disorders 5th edition
DXA: Dual energy X-ray absorptiometry
HRQOL: Health related quality of life
IGT: Iowa Gambling Test
LAGB: Laparoscopic adjustable gastric banding
LRYGB: Laparoscopic Roux-and-Y gastric bypass
LSG: Laparoscopic sleeve gastrectomy
mmHg: Millimeters of mercury
mmol/L: Millimoles per liter
OSA: Obstructive sleep apnea
PedsQL: Pediatric Quality of Life inventory
PFS: Power of Food Scale
SPIRIT: Standard Protocol Items Recommendations for Interventional Trials
SST: Stop Signal Test
T2DM: Type two diabetes mellitus
TBW: Total body water
TFEQ: Three Factor Eating Questionnaire
WLT: Wanting and Liking Test
