# Supplementary material for: Bariatric surgery in adolescents: a prospective randomized controlled trial comparing laparoscopic gastric banding to combined lifestyle interventions in adolescents with severe obesity (BASIC trial)
Source: BMC Pediatr. 2019 Jan 28;19:34. doi: 10.1186/s12887-019-1395-9 (PMC6350363; doi:10.1186/s12887-019-1395-9)
Supplement: Supplementary file 2 — Appendix II: statistical analysis plan for outcome measurements. (DOCX 12 kb) [file 12887_2019_1395_MOESM2_ESM.docx]

**APPENDIX II: STATISTICAL ANALYSIS PLAN FOR OUTCOME MEASUREMENTS**

**Descriptive statistics**

Numerical data will be tested for normality with the Kolmogorov-Smirnov statistics and will be presented as mean ± standard deviation if there is a normal distribution, otherwise as median [range]. Categorical data will be presented as number (percentage).

**Longitudinal treatment effect**

Linear and logistic mixed models will be used to assess the longitudinal treatment effect for numerical and categorical outcome variables, respectively. This analysis method accounts for the correlation between repeated measures, uses all available data, and uses a likelihood approach for missing outcome data, which are assumed to be missing at random (MAR). Group, time and group*time will be included as fixed factors to assess the treatment effect at different time points. Variables related to missing outcome data, based on logistic regression analyses, will be included to the fixed part of the model to ensure MAR. As for the random part, random intercept and/or slope will be considered, where the best option will be selected based on Bayesian Information Criterion (BIC). As sensitivity analysis, baseline characteristics that substantially differ between groups are then also included in the model.

**Interim analysis (if applicable)**

Not applicable.
